# Infantile Monosialoganglioside2 (GM2) Gangliosidosis With Concurrent Bronchopneumonia: An Extraordinary Case of Tay-Sachs Disease

**DOI:** 10.7759/cureus.51797

**Published:** 2024-01-07

**Authors:** Han Grezenko, Shadi S Al-Deir, Filagot D Eshete, Nuzhat Faran, Ciara S Mimms, Muhammad Ibrahim

**Affiliations:** 1 Medicine and Surgery, Guangxi Medical University, Nanning, CHN; 2 Translational Neuroscience, Barrow Neurological Institute, Phoenix, USA; 3 Internal Medicine, Misr University For Science and Technology, Amman, JOR; 4 General Surgery, Jimma University, Jimma, ETH; 5 Internal Medicine, Fatima Memorial Hospital, Lahore, PAK; 6 Medicine, St. George’s University, Great River, USA; 7 Medicine, Jinnah Hospital/Allama Iqbal Medical College, Lahore, PAK

**Keywords:** enzyme replacement therapy, genetic counseling, seizure management, rare pediatric presentation, consanguinity, hexosaminidase a deficiency, neurodegenerative disorders, bronchopneumonia, gm2 gangliosidosis, tay-sachs disease

## Abstract

Tay-Sachs disease (TSD) is a rare, fatal neurodegenerative disorder characterized by the deficiency of the enzyme hexosaminidase-A (Hex A), which results in the accumulation of monosialoganglioside2 (GM2) ganglioside within nerve cells, predominantly affecting individuals of Ashkenazi Jewish descent. We report a remarkable case of a three-year-old South Asian male with infantile GM2 gangliosidosis, compounded by bronchopneumonia, a rarely documented complication in Tay-Sachs patients. The patient presented with recurrent seizures, fever, cough, and developmental delay. Confirmation of the diagnosis was obtained through reduced Hex A enzyme activity, corroborated by imaging and blood and urine analyses. Family history was significant for consanguinity and similar sibling fatalities. Despite the progressive nature of the disease, symptomatic management, including antiepileptic drugs, antibiotic therapy, and supportive care, led to an improvement in clinical condition, though ongoing monitoring remains essential. In this case, the coexistence of bronchopneumonia with Tay-Sachs disease is unusual, reflecting the necessity for this case report. The patient's response highlights the potential for symptomatic management, the importance of genetic counseling, and the imperative for research into gene and enzyme replacement therapies. The uniqueness of this case provides novel insights into the disease's spectrum, enhancing awareness, encouraging early diagnosis, and refining care strategies for Tay-Sachs disease, aligning with the broader goals of improving patient outcomes and advancing medical research.

## Introduction

Tay-Sachs disease (TSD), also known variously as hexosaminidase A (HEXA) deficiency, monosialoganglioside2 (GM2) gangliosidosis type 1, hexosaminidase alpha-subunit deficiency (variant B), B variant GM2 gangliosidosis, and sphingolipidosis Tay-Sachs, is an inherited, devastating neurological disorder. It is characterized by a deficiency of the enzyme hexosaminidase-A, leading to the accumulation of GM2 ganglioside (ganglioside monosialic), a fatty substance, within nerve cells. This accumulation causes progressive neuronal deterioration and severe neurological manifestations [1]. While it is considered a rare condition, Tay-Sachs disease has an estimated incidence of one in 200,000 live births within the general population, with prevalence rates varying among different communities. Certain ethnicities, such as Ashkenazi Jews, exhibit a higher incidence [2].

Due to its rarity, Tay-Sachs disease is the focus of various advocacy groups dedicated to rare diseases. These organizations work closely with researchers, clinicians, and affected families to raise awareness about the disorder and its significant challenges [3].

This case report meticulously documents a clinical encounter with Tay-Sachs disease manifesting in a young child, which is atypical considering the early infantile onset commonly associated with this condition. By presenting this case, we aim to enhance the collective understanding of Tay-Sachs disease's clinical spectrum, contribute to better recognition and diagnosis across diverse age groups, and inform more effective treatment strategies for those afflicted by this debilitating illness.

## Case presentation

**Figure 1:**
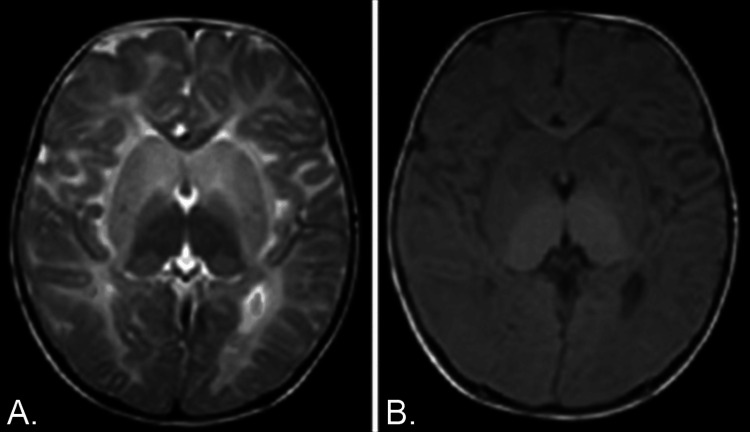
MRI brain images (axial) T2-weighted (T2W) in panel A and T1-weighted (T1W) in panel B MRI images demonstrate symmetrical T2W hypointensity and T1W hyperintensity in the bilateral thalami. Additionally, there is involvement of the bilateral cerebral white matter, which indicates a possible dysmyelination disorder. MRI: Magnetic resonance imaging

A three-year-old male child was brought to the hospital with a recent onset of recurrent tonic-clonic seizures lasting 2-3 minutes, a low-grade intermittent fever, and a non-productive dry cough. The child had a significant past medical history of developmental delays, food refusal, and previous hospital admissions for similar presentations. He was diagnosed with GM2 (ganglioside monosialic) gangliosidosis (Tay-Sachs disease) at a local hospital based on reduced Hex A enzyme activity, blood and urine analyses, and brain MRI findings, along with a family history indicative of a hereditary pattern due to parental consanguinity. MRI brain has been elaborated in Figure 1.

Upon clinical examination, the child was found to have a Glasgow Coma Scale score of 8/15, decreased muscle tone and wasting in the lower limbs, brisk reflexes, and down-going plantar responses. His respiration was rapid and shallow, with crackles noted in the lower zone of the left lung, and ocular twitching was present. These findings suggested a possible respiratory infection.

**Figure 2:**
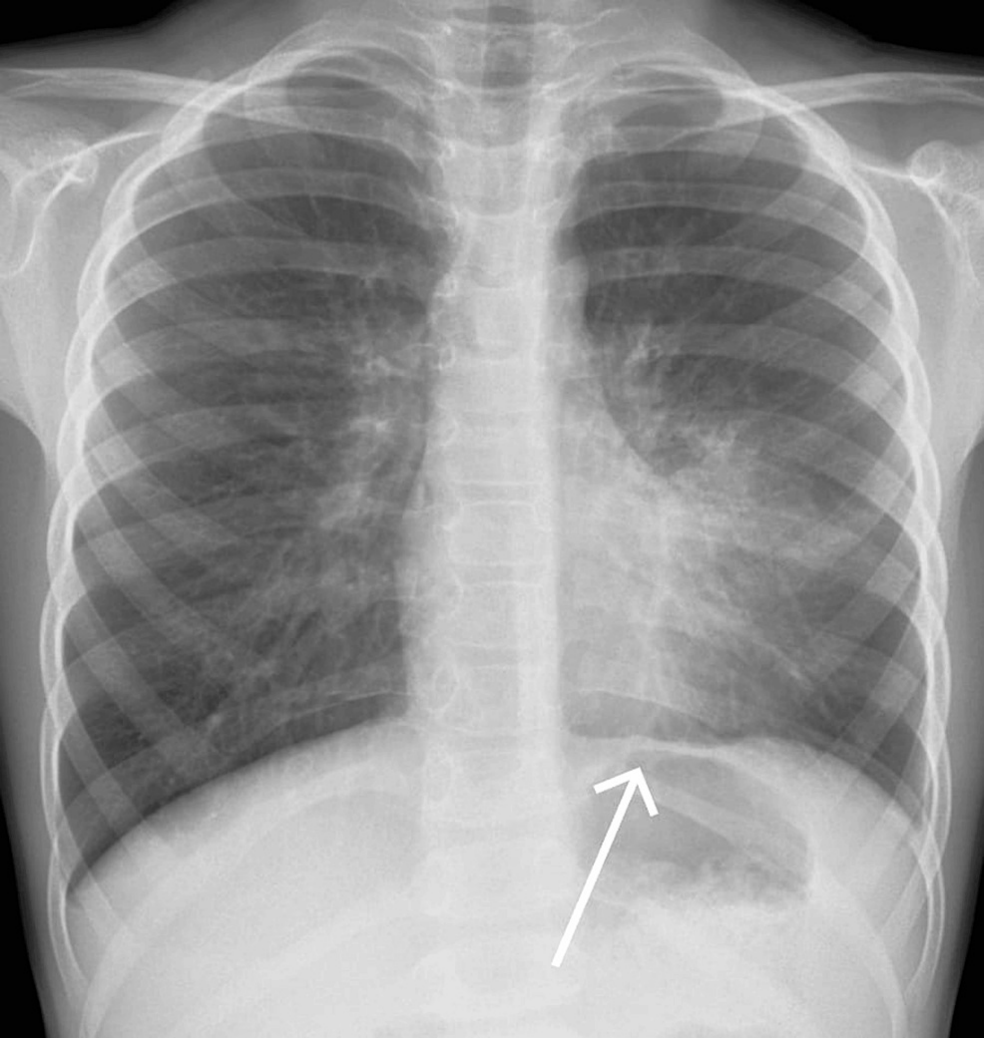
Chest X-ray image The arrow is pointing toward pulmonary opacity on the left lower lobe of the lung.

Laboratory tests, including a complete blood count, renal and liver function tests, and serum electrolytes, were essentially regular, with minor elevations in lymphocyte count and aspartate aminotransferase (AST) levels. Urinalysis showed a turbid appearance with trace proteins. The chest X-ray revealed an opacity in the lower left lung field, indicative of bronchopneumonia. The chest X-ray is shown in Figure 2. A complete blood workup has been provided in Table 1.

**Table 1:** Table representing the complete blood workup of the patient WBC count: white blood cell count, RBC: red blood cells, HCT: hematocrit, MCV: mean corpuscular volume, MCH: mean corpuscular hemoglobin, MCHC: mean corpuscular hemoglobin concentration, ALT: alanine transaminase, AST: aspartate aminotransferase, ALP: alkaline phosphatase.

Hemogram	Patient Value	Reference Range
WBC count	8	4-11 x10^9^/L
Total RBC	4.0	3.8-5.2 x10^12^/L
Hemoglobin	13.6	13-18 (g/dL)
HCT	41	35-46%
MCV	83	77-95 fL
MCH	29	26-32 (pg)
MCHC	34	32-36 (g/dL)
Platelets	433	150-400 x10^9^/L
Neutrophils	38	40-80%
Lymphocytes	56	20-40%
Monocytes	4	2-10%
Eosinophils	2	1-6%
Renal Function Tests		
Urea	22	10-50 mg/dL
Serum Creatinine	0.7	0.5-0.9 mg/dL
Liver Function Tests		
Bilirubin total	0.6	0.3-1.2 mg/dL
Total protein	7.0	5.7-8.2 g/dL
Albumin	4.2	3.2-4.8 g/dL
ALT	37	Up to 40 U/L
AST	90	Up to 40 U/L
ALP	70	40-120 U/L
Serum Electrolytes		
Sodium	138	135-145 mmol/L
Potassium	4.2	3.5-5 mmol/L
Inflammatory Markers		
C-Reactive Protein Quantitative	6	<5

The child's acute presentation was determined to be a combination of his underlying GM2 gangliosidosis with superimposed bronchopneumonia. Treatment was initiated with vigilant bi-hourly monitoring and nasogastric feeding for nutritional support. Chest physiotherapy and postural drainage were employed to manage the respiratory condition. A regimen of intravenous linezolid, Tanzo®, amikacin sulfate, and antiepileptic medications, including topiramate, lamotrigine, and nitrazepam, was administered. After a five-day course of treatment, there was an improvement in the patient's GCS to 11/15, and the antibiotic therapy was slightly de-escalated while maintaining the rest of the treatment.

The child's condition improved significantly throughout the treatment. Yet, he continued to be monitored closely due to the complex nature of his underlying genetic disorder and its interplay with infectious complications. The case highlights the critical need for comprehensive care in managing similar patients with neurodegenerative diseases complicated by common pediatric illnesses.

This confluence of Tay-Sachs disease with bronchopneumonia in a patient of this age, against a backdrop of familial consanguinity, represents a particularly rare and instructive case with significant implications for the management of similar complex pediatric presentations.

## Discussion

Tay-Sachs disease is an autosomal recessive genetic disorder that requires both parents to be carriers for their child to inherit the condition. The disease has a higher prevalence in Ashkenazi Jewish populations, with approximately one in 27 being carriers. In contrast, the general population's carrier rate is about one in 300. Compared to these statistics, Tay-Sachs is exceedingly rare in South Asian populations, where the carrier frequency is estimated to be around one in 250, aligning with the global average [4]. This case is particularly unusual due to the patient's South Asian descent, which adds to its rarity and clinical significance.

Clinically, Tay-Sachs predominantly affects the nervous system. Typical symptoms include seizures, nutritional deficiencies, vision and hearing loss, and respiratory problems [5]. Our patient experienced seizures, a nutritional deficit, and a loss of motor power, along with an atypical complication of bronchopneumonia. The co-occurrence of bronchopneumonia with Tay-Sachs disease is an unusual association and a notable aspect of this case.

There is currently no cure for Tay-Sachs disease; thus, management is directed towards supportive care, including anti-epileptic drugs, antibiotics for concurrent infections, nutritional support, and other measures to prevent complications. Genetic counseling for the parents is also crucial in managing this condition [6]. In the case of our patient, a conventional treatment protocol was administered, augmented with antibiotic therapy for bronchopneumonia and chest physiotherapy.

Public awareness about Tay-Sachs disease and continued research into novel therapeutic interventions is essential. Advances such as gene therapy and enzyme replacement therapy are promising avenues that could transform the management of this disease, making them more broadly available to the public [7].

This case report's significance lies not only in the rarity of Tay-Sachs disease within the South Asian population but also in the presentation of bronchopneumonia as a complicating factor. The discussion around this case underscores the clinical complexity and the need for a multidisciplinary approach to care. Our patient's case exemplifies the challenges faced when a relatively common pediatric condition intersects with a rare genetic disorder, and it emphasizes the importance of considering a broader differential diagnosis. Sharing this case contributes to the body of knowledge required to improve patient care and outcomes for those with similar presentations, making it a worthy addition to the corpus of medical case reports.

An important limitation of this case report warrants mention. While we have strived to provide a comprehensive analysis of this unique case of Tay-Sachs disease, our ability to include a broader range of diagnostic images, particularly those illustrating central nervous system (CNS) reflexes, was constrained. Despite diligent efforts to gather extensive visual data, the availability of such specific images was limited. We acknowledge that additional imaging could have further enriched the understanding of the neurological manifestations in this case. To mitigate this limitation, we have focused on delivering detailed, descriptive accounts of the clinical observations and neurological reflexes observed in the patient. This approach was intended to provide a thorough narrative compensating for the lack of extensive visual documentation. We believe that the detailed textual descriptions offer a substantial understanding of the patient's condition, albeit the visual representation remains an acknowledged limitation of this report.

## Conclusions

This case report presents an atypical occurrence of Tay-Sachs disease, a rare genetic disorder, with concurrent bronchopneumonia in a three-year-old. Despite the progressive and incurable nature of Tay-Sachs, the child showed improvement with a regimen of antiepileptics, antibiotics, and supportive care. Highlighting the necessity for innovative treatments and genetic counseling, this case emphasizes the importance of awareness and research to improve the prognosis and quality of life for those affected by this devastating condition. The report's rarity and the child's unique presentation significantly contribute to the medical literature and patient care strategies.
